# Design, Development, and Characterization of Advanced Textile Structural Hollow Composites

**DOI:** 10.3390/polym13203535

**Published:** 2021-10-14

**Authors:** Zunjarrao Kamble, Rajesh Kumar Mishra, Bijoya Kumar Behera, Martin Tichý, Viktor Kolář, Miroslav Müller

**Affiliations:** 1 Department of Textile & Fiber Engineering, Indian Institute of Technology Delhi, New Delhi 110016, India; ttz178482@textile.iitd.ac.in (Z.K.); B.K.Behera@textile.iitd.ac.in (B.K.B.); 2 Department of Material Science and Manufacturing Technology, Faculty of Engineering, Czech University of Life Sciences Prague, Kamycka 129, 165 00 Prague, Czech Republic; martintichy@tf.czu.cz (M.T.); vkolar@tf.czu.cz (V.K.); muller@tf.czu.cz (M.M.)

**Keywords:** textile structural composite, 3D weaving, hollow structure, spacer fabric, woven honeycomb, sandwich, waste cotton, impact, compression, flexural rigidity

## Abstract

The research is focused on the design and development of woven textile-based structural hollow composites. E-Glass and high tenacity polyester multifilament yarns were used to produce various woven constructions. Yarn produced from cotton shoddy (fibers extracted from waste textiles) was used to develop hybrid preforms. In this study, unidirectional (UD), two-dimensional (2D), and three-dimensional (3D) fabric preforms were designed and developed. Further, 3D woven spacer fabric preforms with single-layer woven cross-links having four different geometrical shapes were produced. The performance of the woven cross-linked spacer structure was compared with the sandwich structure connected with the core pile yarns (SPY). Furthermore, three different types of cotton shoddy yarn-based fabric structures were developed. The first is unidirectional (UD), the second is 2D all-waste cotton fabric, and the third is a 2D hybrid fabric with waste cotton yarn in the warp and glass multifilament yarn in the weft. The UD, 2D, and 3D woven fabric-reinforced composites were produced using the vacuum-assisted resin infusion technique. The spacer woven structures were converted to composites by inserting wooden blocks with an appropriate size and wrapped with a Teflon sheet into the hollow space before resin application. A vacuum-assisted resin infusion technique was used to produce spacer woven composites. While changing the reinforcement from chopped fibers to 3D fabric, its modulus and ductility increase substantially. It was established that the number of crossover points in the weave structures offered excellent association with the impact energy absorption and formability behavior, which are important for many applications including automobiles, wind energy, marine and aerospace. Mechanical characterization of honeycomb composites with different cell sizes, opening angles and wall lengths revealed that the specific compression energy is higher for regular honeycomb structures with smaller cell sizes and a higher number of layers, keeping constant thickness.

## 1. Introduction

Textile structures have shown remarkable performance in advanced composites for aerospace, automotive, marine, civil engineering, wind energy, protective clothing, and many other applications. Unidirectional (UD) and two-dimensional (2D) woven textile-reinforced composites have exhibited clear advantages over the traditional metallic materials in terms of performance-to-weight ratio. Various three-dimensional (3D) woven textile structures have started to receive serious attention for structural composites due to better structural integrity, high delamination resistance, etc. The modern low-cost manufacturing methods of single and multilayer non crimp woven preform have created research interest in these new reinforcement structures [1,2,3,4,5,6]. Modern preform manufacturing technology (weaving, braiding, warp knitting, and nonwoven) also facilitates the development of a variety of complex geometrical shapes [7,8].

In 3D woven fabric-reinforced composites, adjusting parameters of the internal geometry of the preform leads to efficient optimization of the performance of the final product. The fiber orientation in an engineered preform determines the direction of the best possible stiffness and strength performance, while the matrix is responsible for stress transfer and load redistribution in a textile structural composite [9,10,11,12]. The internal architecture of the material governs the mechanical properties of the part and hence offers enormous space for designers to match the ultimate criteria for a specific application. Over and above, modern computational tools help to predict, and hence design, special textile architectures of desired mechanical performance [13,14,15,16,17,18]. Fiber architectures of 3D woven preforms can be adjusted in a wide range by changing the weaving parameters, such as warp/weft density and weaving patterns. The introduction of fiber in thickness direction improves the interlaminar properties. Fiber architectures directly affect the formability of the preforms [19,20,21,22].

An advantage of 3D weaving is that preforms can be made on standard industrial weaving looms used for producing 2D fabrics by making minor modifications to the machinery [23,24,25,26,27,28]. A specialized 3D woven fabric is spacer or distance fabric. This material consists of two parallel 2D woven fabrics integrally connected by a low density of the through-thickness yarns. Spacer fabric composites are an alternative to honeycomb or foam material to make sandwich structures because they exhibit superior mechanical properties [29,30,31,32]. These composites are primarily used to manufacture double-walled tanks or the wall lining for chemical storage tanks, car and truck spoilers/fairings, lightweight walls, dome structures and composite tooling [33]. Sandwich structures constitute a thick and light-weight core sandwiched between two relatively thin face sheets and offer high bending stiffness while being light-weight. Sandwich structures reinforced with integrally produced 3D spacer preforms have very high delamination resistance compared to the conventional sandwich composites [34,35]. The characterization of compressive and bending properties of corrugated core sandwich structures with different core thickness, corrugation angle, and bonding length between core-face sheets have been reported [36,37,38]. In order to produce spacer structures with different cell geometrical parameters, i.e., with different cell wall opening angles and with different cell widths (at almost constant cell heights) and different cell heights (at almost constant cell widths), the required number of picks in different sections of the cross-sections need to be calculated. Using these calculated number of picks, the generalized weave designs for each type of structure can be modified to obtain the actual weave designs [39,40,41]. Sandwich structures with integrated woven core piles have higher skin–core debonding resistance as compared to other sandwich composites [42]. Quasi-static and dynamic compression of such structures demonstrate ductile failure and very good energy-absorbing capability [43]. Increase in the height of core piles reduces the out-of-plane compression load [44], whereas thinner panels exhibit higher absorbed energy per unit volume in quasi-static compressive and three point-bending evaluation [45]. Though these spacer composites are better than traditional sandwich composites in some respect, these structures are not strong enough for flexural loading conditions [46].

In light of the above discussion, intensive research has been carried out to investigate and establish the relative mechanical advantage of some special textile structural composites using a wide range of preform architectures starting from simple chopped fiber to the most complex 3D structures, such as energy-absorbing hollow structures, honeycomb structures, spacers with augmented cores, profiled structures, stiffeners, and aerodynamic structures [47,48]. Woven spacer fabrics with woven cross-links and different cell geometries were produced. The sandwich composites were analyzed for their quasi-static lateral compression and flexural performance to compare their load-bearing capacity and energy absorbency [49,50,51,52,53]. Further, complex profiled 3D fabrics, e.g., I, U, + or X shapes are used in composites where superior joint strength is desired [54,55].

The manufacturing possibilities of woven spacers with woven cross-links have been reported by several researchers. However, to establish end-use based on structural characteristics, it is necessary to study and compare their mechanical behaviors, such as compressive and bending properties. In this research work, sandwich structures with different cell geometrical shapes were manufactured using 3D integrally woven spacer fabrics. These structures were subsequently evaluated for their compressive and flexural performance to reveal their load-bearing capacity, energy absorbency and failure mechanisms.

The current research mainly focuses on several UD, 2D and 3D woven textile hollow structures and profiled structures used in composite reinforcement. Several novel architectures have been designed and developed for applications in aircraft wings, wind turbine blades, etc. The mechanical performance of such hollow composites with respect to their impact, compression and flexural properties were evaluated. Novel sandwich structures were developed by using waste cotton fibers recycled from textile wastes. Hollow structural composites, namely spacer, honeycomb and sandwich, with special geometries were designed for optimal aerodynamic performance. Further, the junction strength of profiled geometries was analyzed. These innovative textile structural composites offer several advantages over chopped fiber or conventional 2D fabric-reinforced composites.

## 2. Materials and Methods

### 2.1. Materials

E-Glass multifilament yarn of 600 tex (Saint-Gobain, Paris, France) was used to produce various woven constructions. The high tenacity polyester multifilament yarn (Reliance Industries Ltd., Mumbai, India) was used to produce sandwich structures with a stiffener section and an augmented core. The physical and mechanical properties of the glass tow and polyester yarn are shown in Table 1. Epoxy resin LY 556 (Sigma-Aldrich Chemicals Private Limited, Bangalore, India) as a matrix and Aradur 22962 (Sigma-Aldrich Chemicals Private Limited, Bangalore, India) as a curing agent was used with a weight ratio of 10:1. The yarn produced from cotton shoddy (fibers extracted from waste textiles) was used to develop homogeneous and hybrid preforms.

### 2.2. Methods

#### 2.2.1. Development of Various Textile Structures

**Figure 1:**
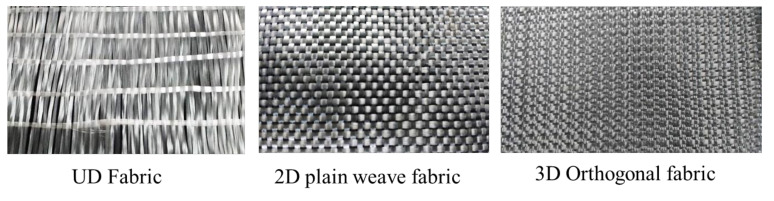
Optical images of UD, 2D and 3D woven preforms.

**Figure 2:**
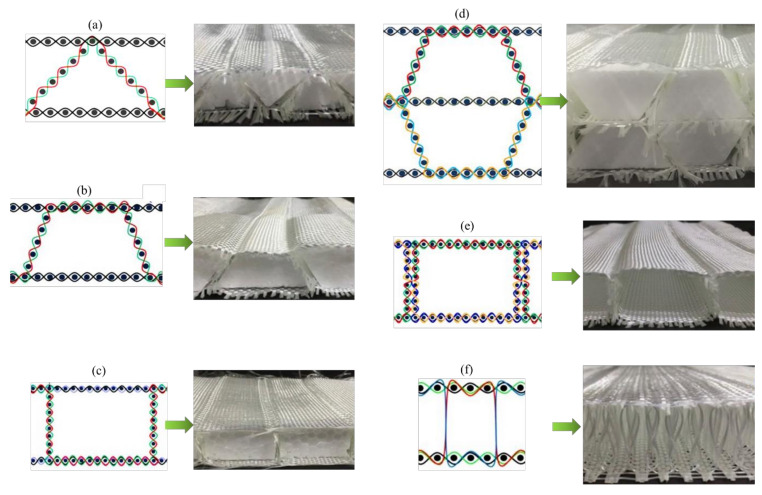
Cross-sectional representation of (**a**) TR, (**b**) TPZ45°, (**c**) RECTSL, (**d**) TDL45°, (**e**) RECTDL, and (**f**) SPY structures.

In this study, unidirectional (UD), two-dimensional (2D) and three-dimensional (3D) fabric preforms were developed. The preform specifications are shown in Table 2. The UD, 2D and 3D fabric preforms were produced using 600 tex E-glass yarn (Figure 1). All the preforms were developed on a sample weaving machine with a multi-beam creel, and the 3D preform weaving technique has been explained in previous studies [5,6]. The spacer fabrics were produced using 600 tex E-glass yarns. The woven spacer fabric preforms with single-layer woven cross-links with four different geometrical shapes, namely rectangular (single wall structure = RECTSL, double wall structure = RECTDL), trapezoidal (single-level structure = TPZ45°, double-level structure = TDL45°), and triangular (TR47°), were produced. The performance of the woven cross-linked spacer structure was compared with the sandwich structure connected with the core pile yarns (SPY). The weaving specifications of spacer structures are shown in Table 3. The cross-sectional representation of different spacer structures is shown in Figure 2.

**Figure 3:**
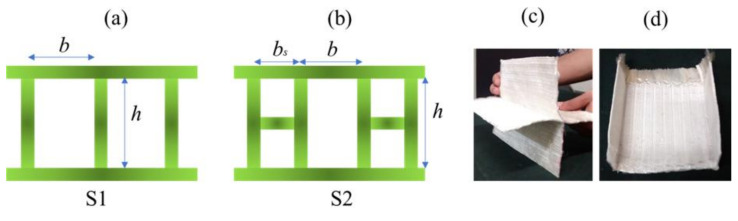
(**a**,**b**) Line diagram showing the geometry of the developed preform structures; (**c**) +-profiled and (**d**) U-profiled integrated woven profiles.

**Figure 4:**
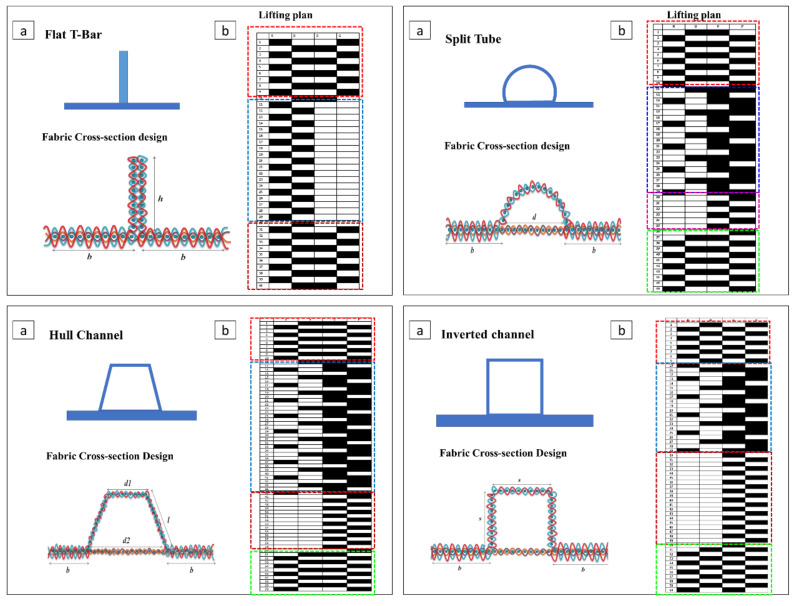
Line diagram of fabric cross-sections and lifting plans of various woven preforms.

**Figure 5:**
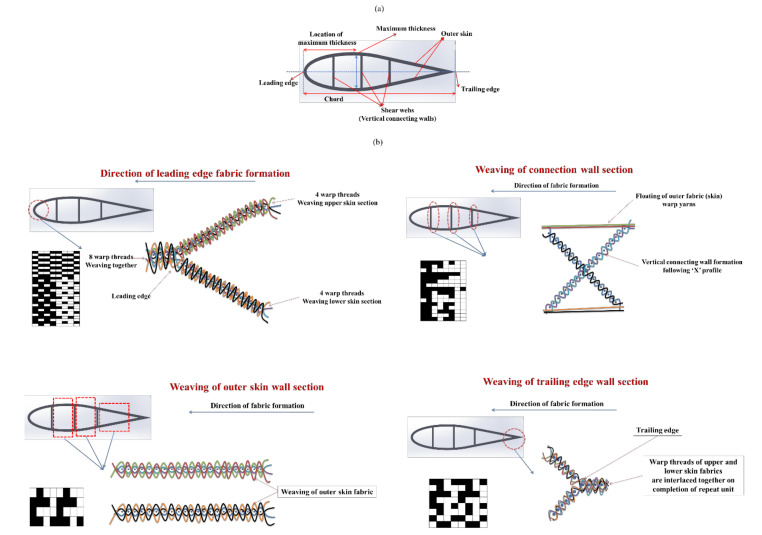
(**a**) Airfoil geometrical attributes; (**b**) development of woven airfoil structure.

Further, an effort to weave sandwich structures with augmented core architecture was developed. Two different sandwich structures of this type were developed (Figure 3a,b). The fabric weaving specifications of these sandwich structures are shown in Table 4. The woven profiles with shapes U and + were produced on the double cloth weaving principle, which has two distinct layers separate from each other (Figure 3c,d). Both the layers of woven fabrics were integrated at a particular point to produce a crucial junction, resulting in an integrated profiled structure. The weaving specifications of these profile structures are shown in Table 5. Four different types of woven preforms (namely split tube, hull channel, inverted channel, and flat T bar) with integrated stiffener sections were developed using modified face to face weaving principle [15]. The line diagrams of these woven preforms and lifting patterns are shown in Figure 4. Based on the same weaving principle, 3D woven aerodynamic spacer structures were developed. The main objective of this invention was to design a woven fabric preform in a similar shape pertaining to a wing profile with the two outer skin sections and shear webs sections integrally woven (connected) so as to be used as reinforcement in developing a one-piece composite wing structure. The geometrical attributes of the airfoil are shown in Figure 5a. Airfoil structures with an ‘I’ and ‘X’ shear web profile were developed. The fabric geometry to be woven is chosen according to the required airfoil shape. The airfoil development process is detailed in Figure 5b. The plain weave was used to weave the preform. The ends and picks per meter were kept at 788 and 394, respectively. The initial information regarding wing profile and its characteristics (airfoil coordinates) was sourced from the NACA (National Advisory Committee for Airfoils) database, which provides the data for the construction of airfoils in the form of points. Each structural element of the 3D spacer structure was converted to an interlacement cross-section with equidistant pick spacing to create a weave design.

**Figure 6:**
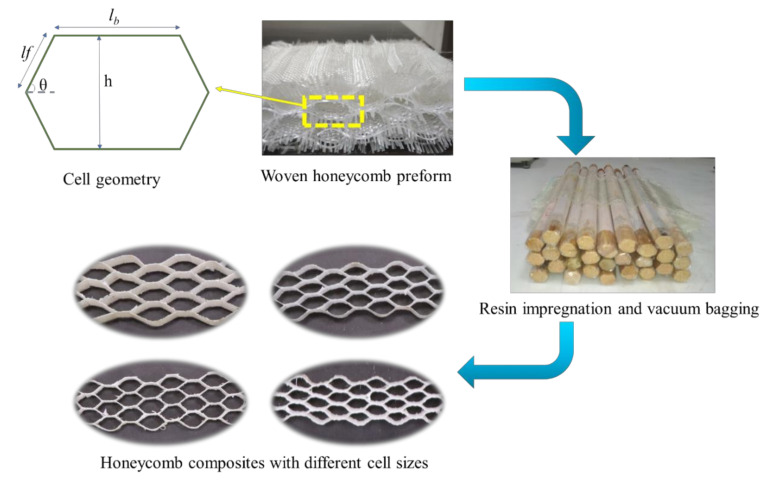
Woven honeycomb preforms, their cell geometry, and composite making process. (Note: *lf* and *l_b_* are the lengths of a free and bonded wall).

Advanced materials based on cellular solids have been used for decades in automotive, marine, and aerospace industries owing to their high energy-absorbing characteristics [18,19]. Metallic honeycombs have been explored as an energy-absorbing cellular structure. However, their strength-to-weight ratio is low, owing to a higher density compared to fiber-reinforced composite materials. E-glass yarn-based woven honeycomb composites with different cell shapes were developed in this research to study their compression properties. The geometrical parameters of the woven honeycomb cell are shown in Figure 6. The honeycomb fabrics with four different cell sizes keeping the opening angle constant were developed (Table 6). The honeycomb fabrics were woven using the double cloth weaving principle [21]. The honeycomb structure is denoted as (x,y)PzLθ, where x is the length of the free wall measured in the number of picks (P), y is the length of bonded measured in the number of picks (P), z is the number of fabric layers used to form a bonded wall, L denotes the layer, and θ is the cell opening angle [21].

Furthermore, three different types of cotton shoddy yarn-based fabric structures were developed. The first is unidirectional (UD), the second is 2D all-waste cotton fabric, and the third is a 2D hybrid fabric with waste cotton yarn in the warp and glass multifilament yarn in the weft. Weaving specifications were decided to achieve approximately the same areal density (Table 7). Four different preform architectures were developed, as follows.

1. Carded cotton shoddy web: The cotton shoddy, which is fibrous material obtained after mechanical shredding of waste cotton fabrics, was treated on a carding machine to produce a fiber web and used as a preform [28].
2. Cotton web sandwiched between woven fabrics: The weight of the preform desired in the composite was calculated based on a relationship between mass, volume, and density. The 2D woven all-waste cotton yarn fabric was cut in line with mold dimensions (30 cm × 30 cm) and weighed. The shoddy web weight was determined by subtracting the woven fabric weight from the total weight of the preform in the composite. The shoddy web has a size in line with mold dimensions. The shoddy web was then sandwiched between woven fabrics during composite fabrication.
3. Cotton web sandwiched between UD preform: this preform was developed by following a procedure similar to preform, with a cotton web sandwiched between woven fabrics. The preform has unidirectional waste cotton yarn fabric at the top and bottom, sandwiching shoddy web.
4. Cotton web sandwiched between hybrid woven fabrics: this preform was developed by following a procedure similar to preform, with a cotton web sandwiched between woven fabrics. The only difference is the hybrid fabric was used as the skin.

#### 2.2.2. Development of Composites Reinforced with Various Textile Structures

The glass tows were chopped to the length of 25 mm uniformly. The stainless-steel mold (30 cm × 30 cm × 0.3 cm) was taken. The chopped glass fibers and matrix weight was calculated according to the relationship between volume, mass, and density, and desired fiber volume in the composite. The fibers were placed in the mold, and resin was applied to them. A uniform application of resin was ensured. The mold was covered with a Teflon sheet and placed in between preheated platens of the compression molding machine. The composites were cured for 60 min. The textile waste-based composite laminates were also developed using a similar methodology. The scheme of the experiment is shown in Table 8, according to which the composite specimens were developed.

**Figure 7:**
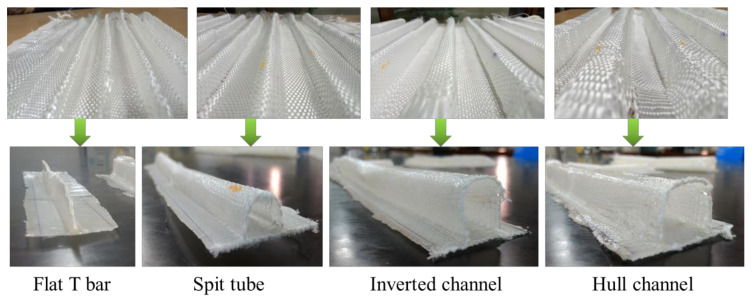
Woven preforms with integrated stiffener sections and their composites.

**Figure 8:**
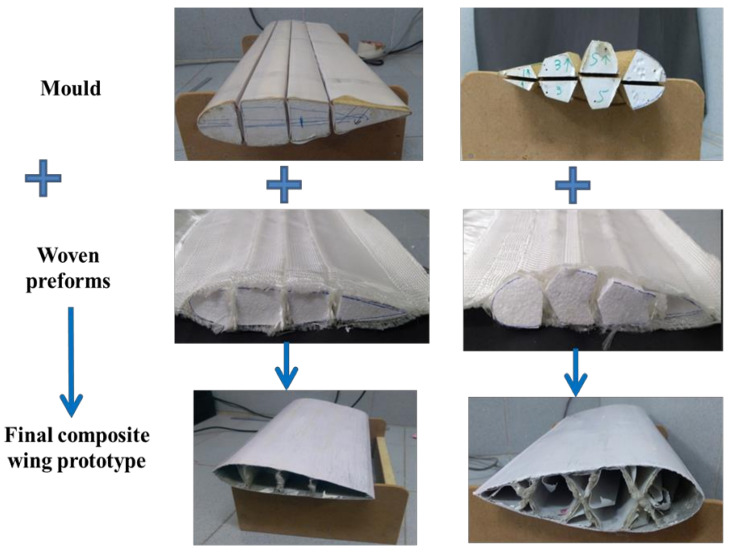
Flowchart for conversion of aerodynamic spacer fabric preforms to composite wing structures.

The UD, 2D and 3D woven fabric-reinforced composites were produced using the vacuum-assisted resin infusion technique. The spacer woven structures were converted to composites by inserting wooden blocks with an appropriate size and wrapped with a Teflon sheet into the hollow space before resin application. A vacuum-assisted resin infusion technique was used to produce spacer woven composites. The samples were cured for 24 h at room temperature. A similar technique was used to produce sandwich composites, composites reinforced with integrated stiffener section, profiled composites, and airfoil structures (Figure 7). The process of woven honeycomb and aerodynamic spacer composite development is depicted in Figure 6 and Figure 8, respectively.

#### 2.2.3. Characterization of Composite Materials

The lateral quasi-static compression and three-point bending of all spacer composites, sandwich composites with augmented core architecture and honeycomb composites were carried out according to ASTM C365 and ASTM C 393, respectively on an Instron 5982 universal testing machine. The junction strength of profiled preforms and their composites was characterized using a universal testing machine in tensile testing mode using a specially designed jaw. The flexural properties of composites reinforced with an integrally woven stiffener section were characterized according to ASTM D 790. The tensile, flexural, and izod impact properties of textile waste-based laminates were characterized according to ASTM D 3039, ASTM D 7264, and ISO 180:2000, respectively.

## 3. Results and Discussion

### 3.1. Mechanical Properties of Composites with UD, 2D and 3D Woven Reinforcement Structures

Three-dimensional woven composites are the materials of choice in many applications, such as aeronautic and astronautic, defense, automotive, construction, safety industry, etc. The fundamental advantage of 3D woven preforms over 2D laminate is the reinforcement in the thickness direction, which holds the yarn layers in place and provides structural stability [33]. This makes 3D woven composites delamination resistant. Further, the 3D weaving technique allows the production of near-net-shape and complex preforms. Three-dimensional woven composites have high tensile strain to failure values, high delamination, and high impact tolerance [34]. Various studies proved that 3D woven preforms produced using natural fiber yarn and their composites have mechanical properties comparable to high-performance fiber-reinforced composites.

**Figure 9:**
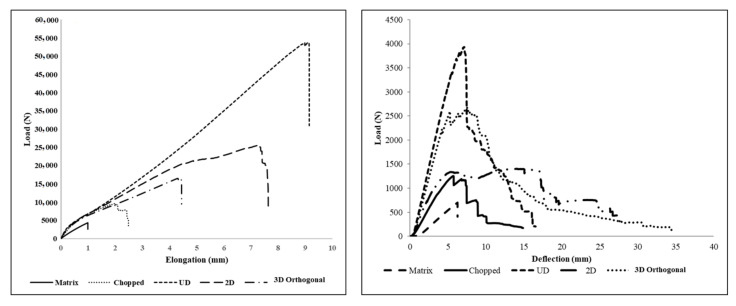
Load-elongation and load-deformation plots of composites reinforced with different reinforcement architecture.

Remarkable improvement in tensile strength and Young’s modulus of textile structure-reinforced composites is observed compared to a neat matrix (Figure 9). While changing the reinforcement from chopped fibers to 3D fabric, its modulus and ductility increase substantially. Tensile test results clearly show that UD fabric-reinforced composite possesses the highest ultimate strength among all other composites. This is due to the higher fiber orientation in the loading direction, followed by 2D fabric-reinforced composite due to comparatively less fiber orientation in the loading direction, while in 3D fabric, reinforced composite fibers are disposed of in three perpendicular planes, leading to lesser strength in warp direction for the given fiber volume fraction and areal density compared to unidirectional reinforced (UD) 2D fabric-reinforced composites. A higher modulus in the composites with UD and 3D preform architecture is a negligible crimp in the warp yarns and zero crimp in the stuffer yarns.

These composites are translucent in nature. Hence, damaged regions of impacted samples become opaque, and internal damage can be visually identified. After testing, a composite’s structural observation reveals that delamination is significantly higher in UD and 2D fabric-reinforced composites. The delamination in 3D is negligible due to through-thickness yarns, which will increase the interlaminar shear strength. The 3D fabric has an integrated architecture compared to all other preforms.

**Figure 10:**
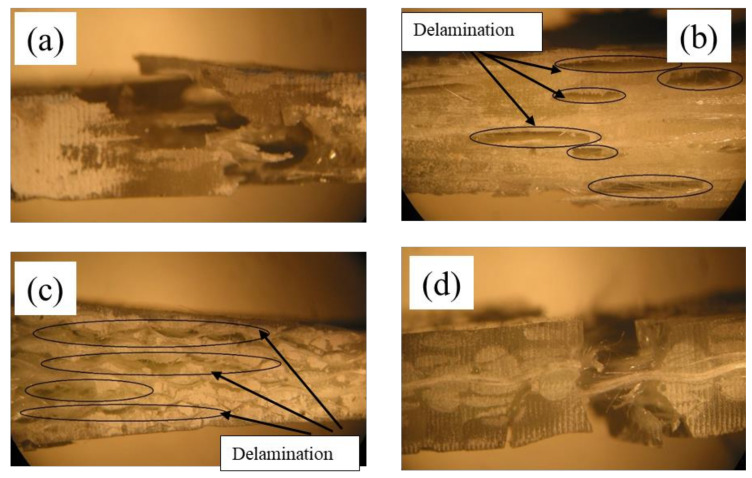
Optical microscope images of the side view of tensile fractured (**a**) chopped fibers; (**b**) UD; (**c**) 2D fabric; and (**d**) 3D orthogonal fabric reinforced composites.

The microscopic analysis (side view) of tensile-tested specimens is shown in Figure 10. The side view near the rupture point of UD fabric layers and 2D fabric layers reinforced composites are shown in Figure 10b,c, respectively. It is clear from the images that delamination is the main reason for the failure of these composites. In these UD and 2D fabric-reinforced composites, the interlaminar connection is only by the matrix. This would form distinctive layers in the composites. When the composites are subjected to tensile loading, the interlaminar shear force will be exerted in the matrix region between the fabric layers. As the matrix has very poor shear strength, it will crack very quickly during loading. This crack in the matrix will increase in the loading direction with an increase in the tensile stress and ultimately lead to the composite’s failure.

In contrast to these two composites, the 3D fabric-reinforced composite has a single integrated fabric in the reinforcement phase. The through-thickness yarns in the Z-direction have higher shear strength compared to the matrix. The microscopic image of tensile fractured 3D orthogonal fabric-reinforced composite is shown in Figure 10d. These two images show the significance of integrated fabric structure in the reinforcement phase. Hence, integrated 3D preform architecture could be majorly preferred for load-bearing applications.

A close observation reveals that a composite’s flexural rigidity reinforced with chopped, 2D and 3D architecture is found to be 60%, 79% and 23% lower than that of a composite with UD fabric reinforcement. Similarly, a composite’s flexural stress reinforced with chopped, 2D and 3D architecture is found to be 67%, 63%, and 25% lower, respectively, compared to a UD-reinforced composite. It indicates that strain energy is highest in UD, followed by 3D, 2D and chopped fiber-reinforced composites. This behavior is mainly because of the orientation of all the tows in the longitudinal direction, and also flexural testing is carried out in the warp direction. However, deflection at break is minimum for this UD composite, whereas in 3D fabric, the reinforced composite shows the second-highest energy absorption with a maximum deflection at the break. The 3D fabric composite shows comparatively less energy absorption than the UD fabric because the tows are oriented in three mutually perpendicular directions.

The maximum deflection in the 3D-reinforced composite during three-point bending is shown in Figure 10. It could be observed from this that the 3D composite can withstand maximum load without a fail in the structure. The opaque region was observed around the ruptured zone indicating the delamination in the composite. As the load is applied in the transverse direction, the composite’s top layer will undergo compression, and the bottom layer undergoes extension. Hence, interlaminar shear force will come into existence between the layers. Due to the poor strength of the matrix, composites reinforced with UD and 2D fabric layers are more prone to delamination than 3D fabric-reinforced composites. The initialization of matrix crack in the composite during flexural testing could be clearly seen from the microscopic images. The 3D fabric-reinforced composite shows a sharp break during transverse loading. This is because of the higher interlaminar strength between the fiber layers in the structure. The higher interlaminar strength is mainly the result of the yarns in the through-thickness direction. Hence, this composite reinforced with 3D fabric is the better choice in the places of load-bearing applications and crashworthiness.

### 3.2. Compressional and Flexural Properties of Sandwich Composites

**Figure 11:**
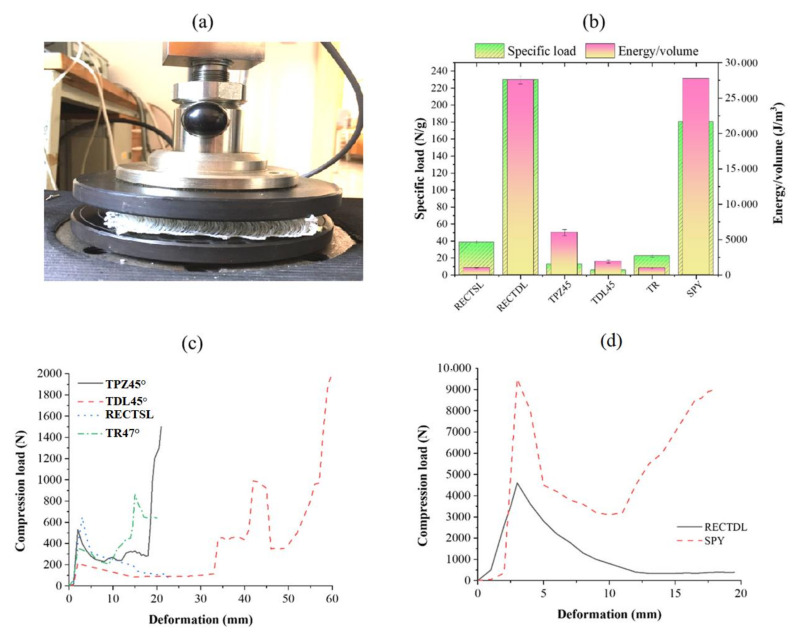
(**a**) Quasi-static compression test, (**b**) specific compressive load and energy/volume of different spacer composites, (**c**,**d**) compression load–deformation curves of different sandwich composites.

The results of the compressive strength of the different composites are shown in Figure 11. It has been observed that the single-wall rectangular spacer structure shows the highest compressive force compared to TPZ and TR. This is due to the angle of load-bearing walls with respect to the direction of applied load. In the case of TPZ and TR, the effective load-carrying capacity of the connective wall reduces from applied load P to Psinθ [38]. In the case of the RECTSL and RECTDL structure, the connecting wall is at a right angle to the face sheet, and therefore it exhibits high load-carrying capacity. However, the SPY structure shows the highest compressive load among all the spacer composites. This is attributed to the uniform distribution of core piles with a density of ~30 piles per square inch. However, in sandwich structures, only two walls take part in load bearing. The compressive strength of the double-wall RECTDL structure is multifold higher than RECTSL due to greater wall thickness. The compressive load-carrying capacity of the double-level TDL45° structure was lower than its single-level structure TPZ45°. Under the applied compressive load, the weak wall buckles sooner than that of the relatively stronger paired wall, which results in a moment at the junction. The mass of the specimen is considered in the calculation of specific compressive strength. However, it does not consider the different volumes of composite specimens, and therefore it cannot be a true representation of compressive performance. Therefore, the strain energy up to maximum compressive load (first peak load) was calculated from the load-deformation curves, and the values were normalized with the volume of the corresponding specimen. The compressional strain energy of the structures was found in order of SPY > RECTDL > TPZ45° > TDL45° > RECTSL > TR. The maximum flexural stress of the sandwich structures was calculated according to the equation below.

$$\mathrm{Maximum}\;\mathrm{flexural}\;\mathrm{stress}\;=\;\frac{F_{max}Ly}{4I}$$

where *Fmax* indicates maximum bending load, *L* is supported span length, *y* is the distance from the neutral axis, and *I* is the area moment of inertia. The flexural stress of the sandwich composites was in the order of RECTDL > RECTSL > TR > TPZ > SPY. The flexural stress of the sandwich composite TDL was lower than its TPZ. In the case of the RECTDL and RECTSL structure, the connecting wall’s alignment with the face sheet is at a right angle, which helps resist the bending deformation. However, in the case of TPZ and TR, the connecting wall is at an angle to the face sheet; thus, the stress experienced by the wall is less than RECTDL and RECTSL. The quasi-static compression test, results and compression force–displacement curves are shown in Figure 11.

### 3.3. Compressional and Flexural Properties of Sandwich Composites with Augmented Core Architecture

**Figure 12:**
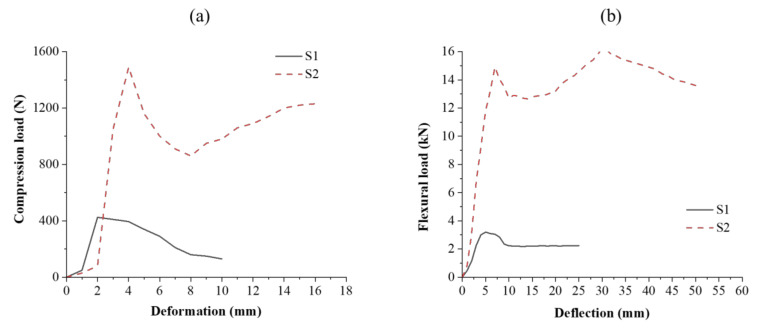
(**a**) Compression load–deformation and (**b**) flexural load–deflection curves of sandwich composites with an augmented core.

Spacer fabrics with vertical connecting walls were selected with an intention to increase the equivalent thickness of their connecting walls (by replacing single connecting walls with double-layered connecting walls) in order to achieve enhanced mechanical performance. Under applied compressive stress, the vertical connecting wall’s destruction, and thus structural deformation, occurs, which leads to core densification. During densification, the core becomes compacted, which indicates that the structure bears load even after core compaction. The peak load varies with core geometry [39]. In structure S1, the single vertical wall buckles or tilts under compressive load. In structure S4, the horizontally integrated section holds the connecting walls from buckling outwards during initial loading, and, therefore, its compressive strength is higher than structure S1 [40,41,42]. The compressional energy of S2 was found to be higher than S1. The compressional resistance is a function of core height, and it decreases with core height. The developed augmented structures exhibited better compressional properties than those of conventional materials [47]. The composites were characterized for flexural properties in three-point bending mode. The bending stiffness of the composite material depends on its elastic modulus, area moment of inertia of the cross-section, and length. The bending stiffness of composite structure S1 was found to be higher than that of S2, which is primarily due to additional load-bearing element in S1. The results have clearly shown that the face sheet acts as a weak point of structure under flexural loading, while its core architecture influences the flexural behavior. Figure 12 shows compression load–deformation and flexural load–deflection curves of sandwich composites with an augmented core.

### 3.4. Flexural Properties of Composites Reinforced with an Integrally Woven Stiffener

**Figure 13:**
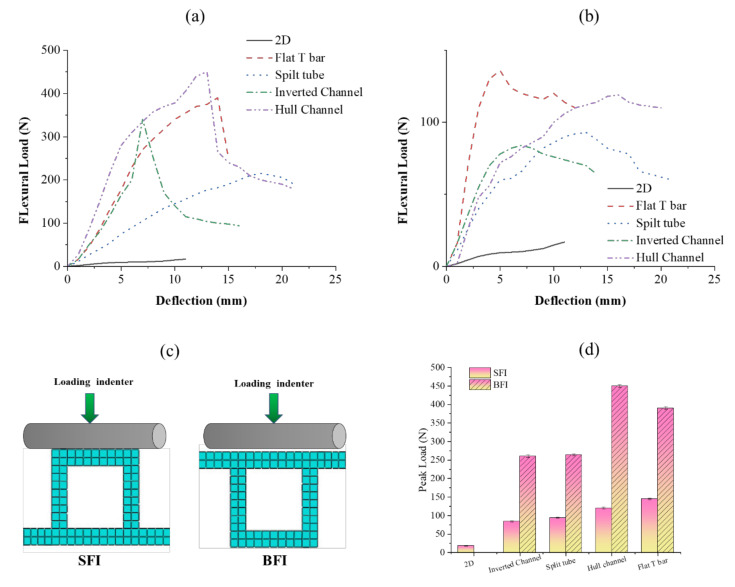
Flexural load–deflection plots of composites with different stiffener sections in (**a**) BFI and (**b**) SFI mode; (**c**) flexural loading configuration of composite samples; and (**d**) comparison of the peak load of different composites.

The developed composites were tested in two modes: (1) stiffener section facing the indenter (SFI), (2) base section facing the indenter (BFI) (Figure 13c). Flexural load–deflection plots are shown in Figure 13a,b. The flexural properties of the developed composites were compared with 2D plain woven polyester fabric-reinforced composites. A higher peak load was observed in BFI mode than SFI. Under the SFI condition, the specimen fails due to the local indentation at the loading point and crippling of the stiffener sections. The stiffener sections, which have higher hollowness, cripple easily and deform under the indenter. A flat T bar with no hollowness exhibits higher flexural load-bearing capacity due to minor crippling and tilting away of stiffener section from the loading axis rather than being structurally deformed. Additionally, only the region of stiffener section which is under the indenter deform during loading and rest structure was observed undeformed. However, a remarkable increment in flexural load-bearing capacity was observed when the specimen was loaded in the BFI condition. This is because BFI condition allows stiffener section to work in coordination with base section, whereas in the SFI condition, the stiffener section deforms quickly. Further, the higher fabric areal density of the base section compared to the stiffener section may also be the reason for the higher flexural carrying capacity in the BFI condition.

Figure 13d shows the peak loads of different stiffened structures characterized for flexural properties under SFI and BFI modes. The 2D fabric-reinforced composite shows little increase in flexural load with an increase in deformation. The peak flexural load of stiffened structures was higher than that of 2D fabric-reinforced composites due to the presence of integrated stiffener. The enhanced flexural performance of stiffened structures is due to an increase in the area moment of inertia of structure during bending. The peak flexural load in the BFI condition was 176, 173, 281, and 200% higher than SFI condition for flat T Bar, split tube, hull channel and inverted channel, respectively.

### 3.5. Junction Strength of Woven U and + Profiled Composites

**Figure 14:**
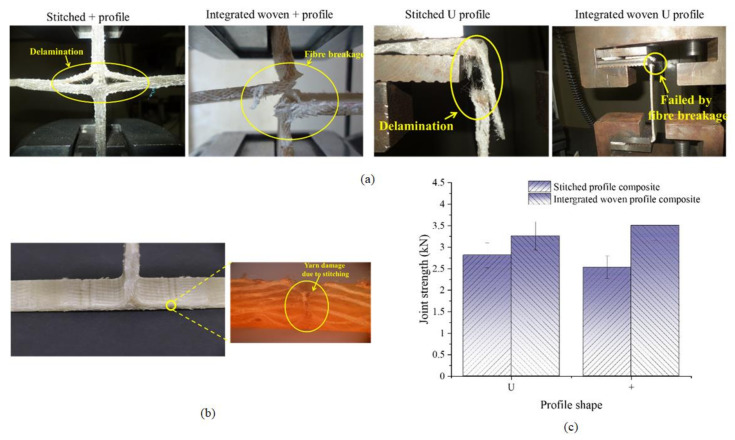
(**a**) Joint strength fractured stitched and integrated woven + and U profiled composite; (**b**) optical microscope image of stitched composite showing yarn damage due to stitching; and (**c**) comparison of joint strength of profiled structures and their composites.

Figure 14c shows the junction strength of U and + woven profiled structures and their composites. The junction strength of integrated woven U and + profiles is 72 and 43% higher than stitched profiles. The stitch line is a stress concentration point in stitched profiles, and the stitching thread is primarily responsible for load bearing at the junction. Additionally, the stitching causes fabric damage due to a higher needle cutting index (Figure 14a) [54]. However, in integrated woven profiles, the yarns within the structure are responsible for junction strength. Further, the junction strength of integrated woven U and + profile composites is 16 and ~39% higher than the junction strength of corresponding stitched profile composites. The stitched structure had a round corner and thick junction area, which results in a high-stress concentration at the junction. Due to the rounded corner, the stitched structure creates a hollow space around it when gripped in the tensile test jaw, which results in less junction strength. The stitched profile composites under tensile load fail when the applied external force exceeds the stitch strength. In this case, the yarn within the composite does not directly take part in load bearing at the junction. The integrated structure has a neat and clean junction with sharp edges. Further, the tensile stress applied on the composite is transferred to the reinforcement through the matrix, and yarns within the integrated woven composite bear the stress. The failure of the integrated woven composite’s joint is primarily due to yarn fracture (Figure 14). Furthermore, it has been observed that the junction strength of stitched U and + profiles after converting them to the composites increases by 81 and 59%, respectively. However, the improvement in the junction strength of integrated woven U and + profiles upon converting to composites is 22 and 54%, respectively.

### 3.6. Drag Force Analysis of Aerodynamic Spacer Structures

**Figure 15:**
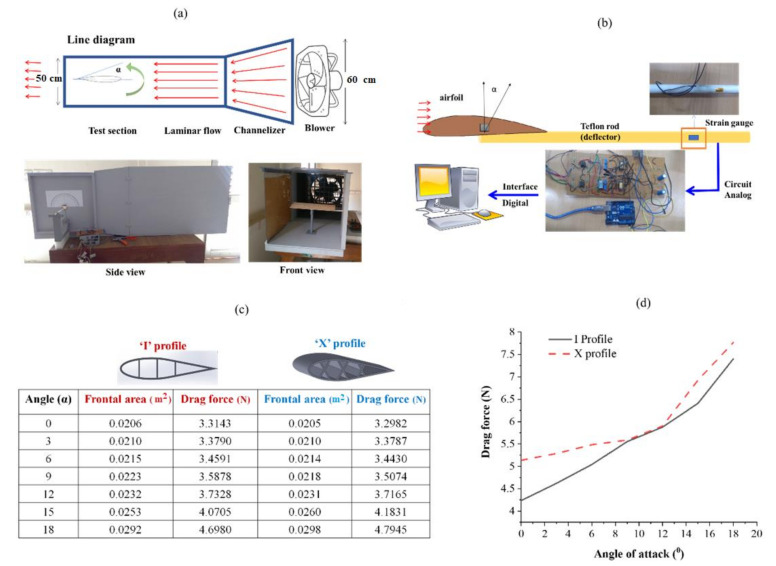
(**a**) Line diagram and actual wing tunnel developed in the laboratory; (**b**) drag force measurement set up installed associated with wind tunnel; (**c**) frontal areas considered, and theoretical drag force calculated at their corresponding angle of attack; (**d**) drag force at a different angle of attack for ‘I’ and ‘X’ profiles.

The flight conditions are assessed using wind tunnel to study aerodynamical efficiency of a prototype aircraft or wing structure. The wind tunnel is used by spacecraft and aircraft making companies namely Boeing, Northrop Gumman, and NASA, etc. The experimental measurement of the drag force generated on the surface of the airfoil was performed using a lab-scale wind tunnel. The measurement was carried out directly using the principle of the cantilever beam deflection (Figure 15a). The drag force measurement setup is described in Figure 15b.

The drag of an object moving in a fluid medium is a function of density, velocity, compressibility and viscosity of the air, the size and shape of the body, and inclination of the body to flow. Therefore, the measurement of drag becomes complex and thus it is necessary to characterize the dependence by a single variable. For drag, this variable is called the drag coefficient (C_d_). The drag (D) is calculated as 0.5C_d_AɤV^2^. Where ɤ and V are density and velocity of air, A is the reference area. The airfoil profile considered in this work is basically the symmetrical airfoils, and the coefficient of drag for a symmetrical airfoil is considered to be around 0.045 from the previous literature. The Area (A) given in the equation refers to the frontal area of the object that is perpendicular to the direction of the fluid flow at a particular angle of attack. The values of density and velocity of the air medium considered for the calculation are 1.223 Kg/m^3^ and 43 m/s, respectively. The corresponding drag force of the wing structures calculated at various angles of attack is tabulated in Figure 15c. For airfoils, at small angles the value of drag is small. With an increase in angle of attack above 5 degrees, the frontal area increases, and thus the boundary layer thickness also increases. The drag force exponentially increases with the angle of attack due to an increase in the frontal area of the wing that tries to resist the flow (Figure 15d).

### 3.7. Compressive Performance of Woven Honeycomb Composites

**Figure 16:**
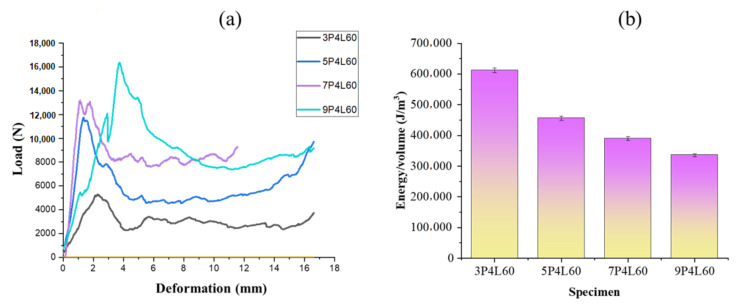
(**a**) Compressive load-deformation plots; and (**b**) energy/volume of honeycomb composites.

The compressive load and energy per unit volume of different honeycomb structures are shown in Figure 16a,b. The compressive load-carrying capacity of the honeycomb composite increases with cell size. This is due to the different sizes of the specimen tested under flatwise compression of the composite. According to ASTM C365, a square shape specimen is required for flatwise compression of honeycomb. The honeycomb cell dimensions increase with a number of picks in the free and bonded wall. Thus, the specimen size increases with cell size, which results in increased load-carrying capacity. However, the strain energy per unit volume decreases linearly with an increase in honeycomb cell size. This is attributed to an increase in specimen volume with honeycomb cell size.

### 3.8. Mechanical Properties of Waste Cotton-Based Composite Laminates

**Figure 17:**
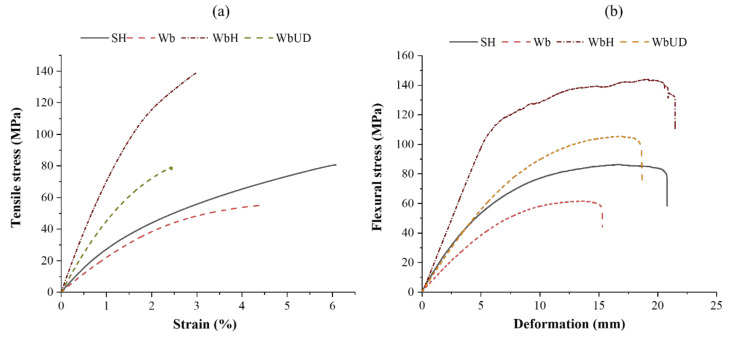
(**a**) Tensile stress–strain curves; and (**b**) flexural stress–deformation curves of composite laminates.

Figure 17a,b shows the tensile stress–strain and flexural stress–deformation plots of textile waste-based composite laminates. It can be observed that the tensile strength and Young’s modulus of composite specimen Wb are 43 and 17%, respectively, lower than SH. The lower tensile strength of composite specimen Wb is due to ~72% of the reinforcement’s total weight within a composite being occupied by woven preform, and ~50% of yarns within the woven preform are not in the loading direction. However, the tensile strength of the WbUD composite is nearly the same as SH due to all the yarns within the skin layers are in the loading direction, and its Young’s modulus is ~73% higher than SH. When tensile stress is applied on the WbUD composite, the outer layer initially bears the stress transferred by the matrix due to its high modulus (unidirectional yarn placement) compared to core material. Upon the outer layer fracture, the load is transferred to cotton fibers at the core, and the complete composite fails when the applied stress exceeds the bearing stress of the cotton fibers at the core. The composite’s tensile strength improves upon stitching due to the enhanced interface between the layers.

The tensile strength and Young’s modulus of the WbH composite is 74% and ~183% higher than the SH composite. This is attributed to high-modulus glass yarn in the loading direction. Further, under the tensile loading of composite specimen WbH, the glass filaments initially bear the stress transferred by the matrix due to its high modulus and low elongation. Upon fracture of glass filaments, the cotton fibers at the core experience the stress transferred by the matrix and fail when the applied stress exceeds its breaking stress. In contrast, tensile strength and Young’s modulus of WbH composite is ~79% and ~63% higher than the WbUD composite.

The flexural strength and flexural modulus of the composite specimen Wb are 40 and 66%, respectively, lower than composite specimen SH. According to sandwich panel theory, when the composite is under three-point bending, the top layer is put into the compressional load, and the bottom into tension, whereas the core is into shear. The laminated composite’s flexural strength and stiffness are controlled by fiber type and its orientation at the composite skin [44,45]. The core is supposed to support the skin to reduce the maximum stress and deformation of the outer layer. The lower flexural strength of composite specimen Wb is due to the early failure of the woven fabric layer at the tension side [46]. However, when UD preform is used at the skin, the flexural strength and modulus of the WbUD composites increase by ~26% and ~74%, respectively, compared to SH. In the case of WbUD, all yarns within the skin layer take part in load-bearing. In contrast, when the 2D woven preform is used as skin, only half of the yarns within the preform take part in bearing tensile load generated at the tension side. The presence of high-strength glass fiber at the skin increases the load-bearing capacity of composite specimen WbH at the tension side, which results in its high flexural strength. The composite specimen WbH has ~68% higher flexural strength than SH.

The impact strength is the energy needed to fracture a composite specimen when subjected to impact loading [47,48]. The izod impact strength of Wb was ~40% higher than composite specimen SH. This was attributed to the high fracture toughness of the cotton yarns present at the skin layer. However, when all yarns within the skin layer are laid unidirectionally, as in composite specimen WbUD, the izod impact strength increases by ~72% than SH. This is due to the increased fracture toughness of the composite skin. The composite specimen WbH shows ~537% higher impact strength than SH. This is attributed to the high fracture toughness of glass filaments present within the skin of composite specimen WbH.

## 4. Conclusions

Fiber reinforced composites have emerged as viable structural materials due to their advantageous stiffness, thermal expansion, strength and density properties. These composites have a high modulus of elasticity, high resistance to fatigue failure, and good resistance to corrosion and they are increasingly used to replace traditional materials such as wood and metals such as steel, iron and aluminum. However, the strength of fiber-reinforced composites in a direction perpendicular to the fibers is extremely low compared with the strength along the length of fibers. The design of components made from these composites is complex and the manufacturing and testing of components are highly specialized. Conventional 2D woven fabrics have several disadvantages regarding the design of certain composite products which include anisotropy, limited conformability, poor in-plane shear resistance, difficulty in handling of open constructions, and reduced yarn to fabric tensile translation efficiency due to yarn crimp and crimp interchange. Three-dimensional weaving, on the other hand, can produce near-net-shaped preforms with complex geometry those are less expensive when converted into composites. Three-dimensional weaving allows the tailoring of properties for specific applications and the composites made out of them show better delamination resistance and damage tolerance, higher tensile strain-to-failure values and high interlaminar fracture toughness properties. Composites reinforced with net-shaped three-dimensional (3D) fabric preforms have emerged as a viable option for parts such as stiffeners and stringers. Three-dimensional weaving also made it possible to develop a wide range of air foils with desired aerodynamic behavior and high crossing strength. The driving forces for using 3D fabrics as reinforcement in composite materials includes the option of using different types of yarns in different directions, flexible fiber orientation and fabric architecture, higher impact tolerance and lower manufacturing costs due to reduced labor intensity in the manufacturing processes. It is established that the number of crossover points in the weave structures offered excellent association with the impact energy absorption and formability behavior which are important for many applications, including automobiles, wind energy, marine and aerospace. Mechanical characterization of 3D woven honeycomb composites with different cell sizes, opening angles and wall lengths revealed that the specific compression energy is higher for regular honeycomb structure with smaller cell sizes and a greater number of layers keeping constant thickness.

## Figures and Tables

**Table 1:** Physical and mechanical properties of E-glass and polyester yarn.

Material	E-Glass Yarn	Polyester Yarn	Waste Cotton Yarn
Linear density (tex)	600	333	476
Density (g/cm^3^)	2.54	1.38	~1.5
Tenacity (gf/den)	5.78	4.88	0.36
Young’s modulus (gf/den)	233	89.94	-
Strain at break (%)	4.5	12.16	15.88

**Table 2:** Specifications of UD, 2D, and 3D woven preforms.

Preform	Unidirectional	2D Plain	3D Orthogonal	3D Angle Interlock	3D Warp Interlock
Stuffer/warp ends/m	708	394	158	98	394
Binder ends/m	-	-	315	492	-
Picks/m	78	275	315	315	275
Stuffer layers	-	-	3	3	-
Fabric thickness (mm)	0.4	0.52	1.45	1.46	1.49
Areal density (kg/m^2^)	0.415	0.414	1.25	1.22	1.26
Fiber volume fraction	0.39	0.31	0.36	0.34	0.34

**Table 3:** Specifications of various woven spacer fabric preforms.

Structure	Cell Opening Angle (deg)	Structure Height (mm)	Top Side Length (mm)	Face Sheet Thickness (mm)	FVF [-]
TPZ45°	45	30	30	0.6	0.46
TDL45°	45	56.8	30	0.9	0.42
RECTSL	90	32	34	0.6	0.46
RECTDL	90	29	50	0.6	0.46
TR47°	47	28	-	0.6	0.47
SPY	-	30	-	0.6	0.40

**Table 4:** Specifications of sandwich structures with an augmented core.

Structure	b (mm)	bs (mm)	h (mm)	Weaving Parameters
S1	45	30	30	Ends/m, Picks/m = 788,472
S2	45	56.8	30	

**Table 5:** Weaving specifications of woven profile structures.

Profile Shape	U	H
Total no of ends	510	720
Jacquard capacity (hooks)	200	400
Ground ends	270	360
Ends/cm, Picks/cm	10,10	10,10
Profile ends	240	360
Reed count	20	20
Denting	5/10	5/10

(Note: profile ends indicate the number of ends required to weave the profile shape, where ground ends indicate the number of the ends needed to weave base structure).

**Table 6:** Structural parameters of woven honeycomb preforms.

Specimen	Bonded Wall Length (mm)	Free Wall Length (mm)	Cell Height (mm)	Opening Angle (°)
3P4L60	7.62	7.62	13.2	60
5P4L60	12.7	12.7	22	60
7P4L60	17.78	17.78	30.8	60
9P4L60	22.86	22.86	39.59	60

**Table 7:** Woven preform specifications.

Preform	Unidirectional	2D Fabric with All Waste Cotton Yarn	2D Hybrid Fabric (Warp—Waste Cotton Yarn, Weft—600 Tex Glass Yarn)
Ends/cm	10	5	5
Picks/cm	-	5	4
Areal density (g/m^2^)	~450	~450	~450

**Table 8:** Scheme of composite laminate development.

Sample ID	Preform Type	Woven Fabric Weight (%) in the Composite	Web/Nonwoven Weight (%) in the Composite	Matrix Weight (%) in the Composite
SH	Carded cotton web	-	38	62
Wb	Cotton web sandwiched between woven fabrics	26.33	10.37	63.3
WbUD	Cotton web sandwiched between waste cotton yarn UD preform	23.24	14.45	62.43
WbH	Cotton web sandwiched between hybrid woven fabrics	26.79	8.93	64.28

## Data Availability

Not applicable.

## References

[1] Bannister M.K. (2004). Development and application of advanced textile composites. Proc. Inst. Mech. Eng. Part L J. Mater. Des. Appl..

[2] Karaduman N.S., Karaduman Y., Ozdemir H., Ozdemir G. (2017). Textile Reinforced Structural Composites for Advanced Applications. Textiles for Advanced Applications.

[3] Behera B.K., Dash B.P. (2015). Mechanical behavior of 3D woven composites. Mater. Des..

[4] Patel D.K., Waas A.M., Yen C.F. (2019). Compressive response of hybrid 3D woven textile composites (H3DWTCs): An experimentally validated computational model. J. Mech. Phys. Solids.

[5] Dash A.K., Behera B.K. (2019). Weave design sspects of 3D textile preforms towards mechanical properties of their composites. Fibers Polym..

[6] Patel D.K., Waas A.M., Yen C.F. (2018). Direct numerical simulation of 3D woven textile composites subjected to tensile loading: An experimentally validated multiscale approach. Compos. Part B Eng..

[7] Mishra R., Gupta N., Pachauri R., Behera B.K. (2015). Modelling and simulation of earthquake resistant 3D woven textile structural concrete composites. Compos. Part B Eng..

[8] Moshtaghian Z., Hasani H., Zarrebini M., Shirazi M.P. (2020). Development and auxetic characterization of 3D composites produced with newly-designed multi-cell flat-knitted spacer fabrics. J. Ind. Text..

[9] Bunzel F., Wisner G., Stammen E., Dilger K. (2020). Structural sandwich composites out of wood foam core and textile reinforced concrete sheets for versatile and sustainable use in the building industry. Mater Today Proc..

[10] Manjunath R.N., Behera B.K., Mawkhlieng U. (2019). Flexural stability analysis of composite panels reinforced with stiffener integral woven preforms. J. Text. Inst..

[11] Torre L., Kenny J.M. (2000). Impact testing and simulation of composite sandwich structures for civil transportation. Compos. Struct..

[12] Shin K.B., Lee J.Y., Cho S.H. (2008). An experimental study of low-velocity impact responses of sandwich panels for Korean low floor bus. Compos. Struct..

[13] Tripathi L., Neje G., Behera B.K. (2020). Geometrical modeling of 3D woven honeycomb fabric for manufacturing of lightweight sandwich composite material. J. Ind. Text..

[14] Li D., Zhao C., Jiang N., Jiang L. (2015). Fabrication, properties and failure of 3D integrated woven spacer composites with thickened face sheets. Mater. Lett..

[15] Li D., Zhao C., Jiang L., Jiang N. (2014). Experimental study on the bending properties and failure mechanism of 3D integrated woven spacer composites at room and cryogenic temperature. Compos. Struct..

[16] Kamble Z., Behera B.K., Mishra R., Behera P.K. (2021). Influence of cellulosic and non-cellulosic particle fillers on mechanical, dynamic mechanical, and thermogravimetric properties of waste cotton fiber reinforced green composites. Compos. Part B Eng..

[17] Mouritz A.P., Bannister M.K., Falzon P.J., Leong K.H. (1999). Review of applications for advanced three-dimensional fiber textile composites. Compos. Part A Appl. Sci. Manuf..

[18] Neje G., Behera B.K. (2019). Investigation of mechanical performance of 3D woven spacer sandwich composites with different cell geometries. Compos. Part B Eng..

[19] Pirouzfar S., Zeinedin A. (2021). Effect of geometrical parameters on the flexural properties of sandwich structures with 3D-printed honeycomb core and E-glass/epoxy Face-sheets. Structures.

[20] Song S., Xiong C., Zheng J., Yin J., Zou Y., Zhu X. (2021). Compression, bending, energy absorption properties, and failure modes of composite Kagome honeycomb sandwich structure reinforced by PMI foams. Compos. Struct..

[21] Manjunath R.N., Khatkar V., Behera B.K. (2020). Influence of augmented tuning of core architecture in 3D woven sandwich structures on flexural and compression properties of their composites. Adv. Compos. Mater..

[22] Fan H., Zhou Q., Yang W., Jingjing Z. (2010). An experiment study on the failure mechanisms of woven textile sandwich panels under quasi-static loading. Compos. Part B Eng..

[23] Zhao C., Li D.S., Ge T.Q., Jiang L., Jiang N. (2014). Experimental study on the compression properties and failure mechanism of 3D integrated woven spacer composites. Mater. Des..

[24] Vuure A.W., Ivens J.A., Verpoest I. (2000). Mechanical properties of composite panels based on woven sandwich-fabric preforms. Compos. Part A Appl. Sci. Manuf..

[25] Karahan M., Ulcay Y., Eren R., Karahan N., Kaynak G. (2010). Investigation into the tensile properties of stitched and unstitched woven Aramid/Vinyl Ester composites. Text. Res. J..

[26] Ahmed K.S., Vijayarangan S. (2008). Tensile, flexural and interlaminar shear properties of woven jute and jute-glass fabric reinforced polyester composites. J. Mater. Process. Technol..

[27] Kamble Z., Behera B.K., Kimura T., Haruhiro I. (2020). Development and characterization of thermoset nanocomposites reinforced with cotton fibers recovered from textile waste. J. Ind. Text..

[28] Lee H., Kureemun U., Ravandi M., Teo W.S. (2020). Performance of interlaminar flax-carbon hybrids under bending. Procedia Manuf..

[29] Jawaid M., Abdul Khalil H.P.S., Abu Bakar A. (2010). Mechanical performance of oil palm empty fruit bunches/jute fibers reinforced epoxy hybrid composites. Mater. Sci. Eng. A.

[30] Dau F., Dano M.L., Vérone B., Girardot J., Aboura Z., Morvan J.M. (2021). In-plane and out-of-plane characterization of a 3D angle interlock textile composite. Compos. Part A Appl. Sci. Manuf..

[31] Liu T., Fan W., Wu X. (2019). Comparisons of influence of random defects on the impact compressive behavior of three different textile structural composites. Mater. Des..

[32] Ladani R.B., Wang C.H., Mouritz A.P. (2019). Delamination fatigue resistant three-dimensional textile self-healing composites. Compos. Part A Appl. Sci. Manuf..

[33] Wondmagegnehu B.T., Paramasivam V., Selvaraj S.K. (2021). Fabricated and analyzed the mechanical properties of textile waste/glass fiber hybrid composite materiál. Mater. Today Proc..

[34] Zhang J., Zhang W., Huang S., Gu B. (2021). An experimental–numerical study on 3D angle-interlock woven composite under transverse impact at subzero temperatures. Compos. Struct..

[35] Flores-Johnson E.A., Li Q.M. (2011). Experimental study of the indentation of sandwich panels with carbon fibre-reinforced polymer face sheets and polymeric foam core. Compos. Part B Eng..

[36] Conejos F., Balmes E., Tranquart B., Monteiro E., Martin G. (2021). Viscoelastic homogenization of 3D woven composites with damping validation in temperature and verification of scale separation. Compos. Struct..

[37] Yan S., Zeng X., Long A. (2020). Effect of fibre architecture on tensile pull-off behaviour of 3D woven composite T-joints. Compos. Struct..

[38] Jiao W., Chen L., Xie J., Yang Z., Fang J., Chen L. (2020). Effect of weaving structures on the geometry variations and mechanical properties of 3D LTL woven composites. Compos. Struct..

[39] Ruggles-Wrenn M.B., Alnatifat S.A. (2021). Fully-reversed tension-compression fatigue of 2D and 3D woven polymer matrix composites at elevated temperature. Polym. Testing.

[40] Nayak S.Y., Shenoy B.S., Sultan M.T.B., Kini C.R., Shenoy K.R., Acharya A., Jaideep J.R. (2021). Influence of stacking sequence on the mechanical properties of 3D E-glass/bamboo non-woven hybrid epoxy composites. Mater. Today Proc..

[41] Li Z., Li D., Zhu H., Guo Z., Jiang L. (2020). Mechanical properties prediction of 3D angle-interlock woven composites by finite element modeling method. Mater. Today Commun..

[42] Guo Q., Zhang Y., Guo R., Ma M., Chen L. (2020). Influences of weave parameters on the mechanical behavior and fracture mechanisms of multidirectional angle-interlock 3D woven composites. Mater. Today Commun..

[43] Zeng C., Liu L., Bian W., Leng J., Liu Y. (2021). Bending performance and failure behavior of 3D printed continuous fiber reinforced composite corrugated sandwich structures with shape memory capability. Compos. Struct..

[44] Zheng T., Li S., Wang G., Hu Y., Zhao C. (2022). Mechanical and energy absorption properties of the composite XX-type lattice sandwich structure. Eur. J. Mech.-A/Solids.

[45] Chene D., Yan R., Lu X. (2021). Mechanical properties analysis of the naval ship similar model with an integrated sandwich composite superstructure. Ocean Eng..

[46] Reddy C.N., Rajeswari C., Malyadri T., Hari S.N.S. (2021). Effect of moisture absorption on the mechanical properties of jute/glass hybrid sandwich composites. Mater. Today Proc..

[47] Khalili S., Khalili S.M.R., Farsani R., Mahajan P. (2020). Flexural properties of sandwich composite panels with glass laminate aluminum reinforced epoxy facesheets strengthened by SMA wires. Polym. Testing.

[48] Flores-Johnson E.A., Li Q.M. (2012). Structural behaviour of composite sandwich panels with plain and fibre-reinforced foamed concrete cores and corrugated steel faces. Compos. Struct..

[49] Caglayan C., Gurkan I., Gungor S., Cebeci H. (2018). The effect of CNT-reinforced polyurethane foam cores to flexural properties of sandwich composites. Compos. Part A Appl. Sci. Manuf..

[50] Bharath H.S., Bonthu D., Gururaj S., Prabhakar P., Doddamani M. (2021). Flexural response of 3D printed sandwich composite. Compos. Struct..

[51] Nanayakkara A., Feih S., Mouritz A.P. (2011). Experimental analysis of the through-thickness compression properties of z-pinned sandwich composites. Compos. Part A Appl. Sci. Manuf..

[52] Mishra R., Huang J., Kale B., Zhu G., Wang Y. (2014). The production, characterization and applications of nanoparticles in the textile industry. Text. Prog..

[53] Wei X., Wu Q., Gao Y., Yang Q., Xiong J. (2022). Composite honeycomb sandwich columns under in-plane compression: Optimal geometrical design and three-dimensional failure mechanism maps. Eur. J. Mech.-A/Solids.

[54] Hassanzadeh S., Hasani H., Zarrebini M. (2016). Thermoset composites reinforced by innovative 3D spacer weft-knitted fabrics with different cross-section profiles: Materials and manufacturing process. Compos. Part A Appl. Sci. Manuf..

[55] Guo J., Wen W., Zhang H., Cui H. (2021). Warp-loaded mechanical performance of 3D orthogonal layer-to-layer woven composite perforated structures with different apertures. Compos. Struct..

